# Association between C-reactive protein to triglyceride-glucose index and atrial fibrillation: a cross-sectional analysis

**DOI:** 10.3389/fendo.2026.1790373

**Published:** 2026-03-11

**Authors:** JingShuang Liu, Shuhan Pan, Bingxue Song, Xinren Zhang, Xia Li, Jing Li

**Affiliations:** 1 Department of Emergency Medicine, The Affiliated Hospital of Qingdao University, Qingdao, China; 2 Department of Cardiology, The Affiliated Hospital of Qingdao University, Qingdao, China; 3 Department of Biostatistics, School of Public Health, Cheeloo College of Medicine, Shandong University, Jinan, China

**Keywords:** atrial fibrillation, C-reactive protein, cross-sectional study, inflammation, insulin resistance, triglyceride-glucose index

## Abstract

**Objective:**

This study aimed to investigate the association between a novel composite biomarker, the C-reactive protein to triglyceride-glucose index (CTI) and the prevalence of atrial fibrillation (AF) in a general adult population.

**Methods:**

This cross-sectional study included 2, 988 participants Clinical and laboratory data were collected to calculate the CTI. Multivariable logistic regression models were employed to evaluate the association between CTI and AF prevalence, and the dose–response relationship was assessed using restricted cubic spline (RCS) analysis. Furthermore, the association between CTI and AF risk was evaluated using the C-index, net reclassification improvement (NRI), and integrated discrimination improvement (IDI).

**Results:**

Among the 2, 988 participants, CTI levels were significantly higher in the AF group compared to the non-AF group. In the fully adjusted model (adjusting for age, sex, BMI, blood pressure, glycemic indices, and lipid profiles), CTI was independently and positively associated with AF prevalence (OR 7.27, 95% CI 5.87–9.00; P < 0.001). Restricted cubic spline analysis revealed a significant linear dose-response relationship between CTI and AF risk. Furthermore, adding CTI to the reference clinical model improved the C-index from 0.6828 to 0.7985, accompanied by significant improvements in the net reclassification improvement (NRI: 0.5609) and integrated discrimination improvement (IDI: 0.7182) (all P < 0.001).

**Conclusions:**

A higher CTI, reflecting a state of combined elevated inflammation and insulin resistance, is significantly and independently associated with an increased prevalence of atrial fibrillation. This novel index may serve as a valuable and integrative biomarker for identifying individuals at high risk for AF.

## Introduction

Atrial fibrillation (AF) is the most common sustained cardiac arrhythmia encountered in clinical practice (1, 2). With ongoing changes in lifestyle and dietary patterns, together with the rapid aging of the global population, the incidence of AF has increased dramatically worldwide (3). AF is associated with a substantially elevated risk of ischemic stroke, heart failure, and cognitive impairment, and imposes a considerable burden on health care systems and society (4). Therefore, early identification of individuals at high risk and timely intervention targeting modifiable risk factors are essential strategies for controlling AF related risk and preventing disease progression.

Abnormal glucose metabolism and insulin resistance (IR) play important roles in atrial electrical remodeling and structural remodeling, thereby contributing to the development of AF (5–7). The triglyceride–glucose (TyG) index, a novel surrogate marker of IR that integrates fasting triglyceride and glucose levels to quantify metabolic dysfunction, has been shown to be closely associated with an increased risk of AF (8). Inflammation represents another key mechanism in the initiation and progression of AF (9). C-reactive protein (CRP) is a commonly used biomarker of systemic inflammation and has been linked to both the pathophysiology and prognosis of AF (10). However, CRP as a single biomarker has inherent limitations. Although CRP reliably reflects systemic inflammatory status, inflammation and metabolic dysregulation frequently coexist and interact in a bidirectional manner. As a result, a single marker may be insufficient to capture the complex pathophysiological background underlying AF.

The C-reactive protein–triglyceride–glucose index (CTI) is a novel composite indicator that integrates CRP and the TyG index, aiming to simultaneously assess chronic inflammation and insulin resistance within a single metric (11). Compared with individual biochemical markers, CTI may more comprehensively reflect the synergistic and interactive effects between inflammatory stress and metabolic dysfunction (12, 13). Accordingly, the present study aimed to investigate the association between CTI and AF, with the goal of providing a simple and integrated clinical tool that incorporates both inflammatory and metabolic dimensions for early AF risk screening.

## Methods

### Study design and population

This cross-sectional study was conducted at the Affiliated Hospital of Qingdao University using data extracted from the electronic medical records (EMR). Physical examination findings and laboratory measurements were obtained from routine clinical assessments. Sociodemographic characteristics, health status, and lifestyle factors were extracted from the EMRs, as previously described (14).

The study design and protocol received written approval from the Institutional Review Boards of the affiliated hospital of Qingdao University (QYFY WZLL 28911). Individual informed consent was not required as we utilized anonymized data sourced from EMRs. All methods were performed in accordance with the Strengthening the Reporting of Observational Studies in Epidemiology (STROBE) guidelines.

### Inclusion and exclusion criteria

The inclusion criteria for this study were as follows: (1) adult participants aged ≥18 years; (2) availability of complete baseline anthropometric and laboratory data; and (3) a confirmed diagnosis of AF in clinical records based on the International Classification of Diseases 10th Revision, (ICD-10) code I48. Those without such a diagnosis served as controls.

**Figure 1:**
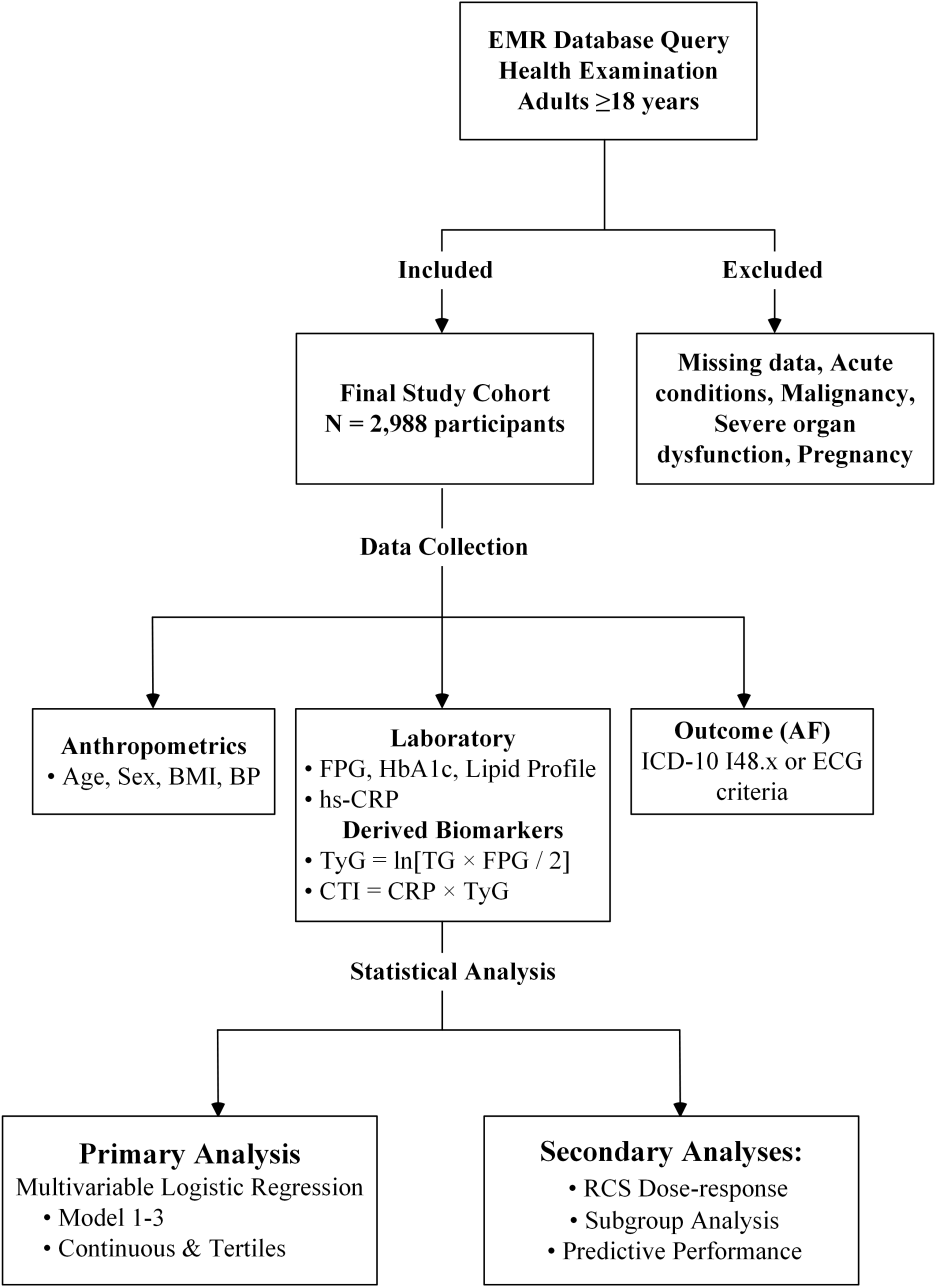
Flow chart of study.

Patients were excluded from the study if they met any of the following criteria: (1) missing key laboratory or clinical data; (2) presence of acute infectious or inflammatory diseases at the time of examination, malignant tumors, severe hepatic or renal dysfunction, or pregnancy; and (3) individuals with incomplete diagnostic information or implausible laboratory values. After applying the inclusion and exclusion criteria, a total of 2, 988 participants were included in the final analysis (As shown in **Figure 1** for the participant flow chart).

### Baseline anthropometric and laboratory measurements

Anthropometric data, including age, sex, height, weight, and blood pressure (SBP and DBP), were collected for all participants. Body mass index (BMI) was calculated as weight in kilograms divided by the square of height in meters. Laboratory measurements included fasting plasma glucose (FPG), glycated hemoglobin (HbA1c), and a lipid profile comprising total cholesterol (TC), triglycerides (TG), low-density lipoprotein cholesterol (LDL-C), and high-density lipoprotein cholesterol (HDL-C). Additionally, CRP levels were measured as a marker of systemic inflammation using standard laboratory assays (14).

### Definitions

Definition of AF: AF was identified either using the ICD-10-CM codes I48.x or based on electrocardiogram findings, characterized by the absence of discrete P waves and the presence of irregularly irregular R-R intervals on a 12-lead EKG or a 24-hour Holter monitoring (14).

Definition of TyG Index: The TyG index, a surrogate marker of IR, was calculated by integrating fasting TG and FPG levels to quantify metabolic dysfunction. TyG = ln [TG(mg/dL) × FPG(mg/dL)/2] (15).

Definition of CTI: The CTI is a novel composite biomarker constructed by integrating CRP and the TyG index, aiming to simultaneously evaluate systemic chronic inflammation and metabolic dysregulation within a single metric. CTI= CRP × TyG.

### Statistical analysis

Continuous variables are reported as mean ± SD and categorical variables as n (%). Groups were compared using t tests or chi square tests, as appropriate. The CTI was modeled as a continuous variable and by tertiles (T1 as reference). Multivariable logistic regression estimated odds ratios (ORs) and 95% confidence intervals (CIs) for atrial fibrillation: unadjusted (Model 1), adjusted for age, sex, and BMI (Model 2), and further adjusted for SBP, DBP, HbA1c, LDL-C, HDL-C, and total cholesterol (Model 3). Trends across tertiles were tested using tertile medians. Subgroup analyses were performed with interaction terms in Model 3. Restricted cubic splines assessed dose response. Discrimination were evaluated using the C index, net reclassification improvement, and integrated discrimination improvement. Analyses were performed in R; two sided P < 0.05 was considered significant.

## Results

### Baseline characteristics of participants stratified by atrial fibrillation

As **Table 1** shown, a total of 2, 988 participants were enrolled in this study, comprising 1, 050 patients with AF and 1, 948 non-AF participants. Participants with AF were older than those without AF (75.84 ± 10.74 vs 68.17 ± 11.36 years, P < 0.001). The AF group also had higher BMI (33.30 ± 7.70 vs 32.01 ± 7.66 kg/m², P < 0.001), higher SBP and DBP (SBP: 133.87 ± 10.49 vs 132.05 ± 12.29 mmHg; DBP: 75.03 ± 6.34 vs 71.75 ± 7.08 mmHg; both P < 0.001), and worse glycemic indices (FPG: 7.03 ± 1.16 vs 6.66 ± 1.19 mmol/L; HbA1c: 7.44 ± 1.43% vs 7.10 ± 1.35%; both P < 0.001). LDL-C was higher and HDL-C was lower among participants with AF (LDL-C: 2.45 ± 0.74 vs 2.25 ± 0.74 mmol/L; HDL-C: 1.10 ± 0.32 vs 1.17 ± 0.31 mmol/L; both P < 0.001). Total cholesterol was lower in the AF group (4.03 ± 0.94 vs 4.32 ± 0.89 mmol/L, P < 0.001), while triglycerides did not differ between groups (P = 0.495). CRP was higher in the AF group (2.88 ± 1.16 vs 2.25 ± 1.13 mg/L, P < 0.001). The AF group had a higher proportion of males (53.14% vs 41.79%, P < 0.001). Antihypertensive medication and aspirin use were more common in AF (both P < 0.001), whereas statin use differed between groups (P = 0.001). Insulin use was not significantly different (P = 0.81). CTI distribution differed substantially by AF status, with 54.86% of AF participants in the highest CTI tertile versus 21.71% in the non-AF group (P < 0.001).

**Table 1:** Baseline characteristics of participants stratified by atrial fibrillation.

Variable	Overall	Non-AF	AF	*P value*
n	2988	1948	1050	
**Age, years, mean (SD)**	70.85 (11.73)	68.17 (11.36)	75.84 (10.74)	<0.001
**BMI, kg/m², mean (SD)**	32.85 (7.71)	33.30 (7.70)	32.01 (7.66)	<0.001
**SBP, mmHg, mean (SD)**	133.23 (11.18)	133.87 (10.49)	132.05 (12.29)	<0.001
**DBP, mmHg, mean (SD)**	73.88 (6.79)	75.03 (6.34)	71.75 (7.08)	<0.001
**FPG, mmol/L, mean (SD)**	6.79 (1.20)	6.66 (1.19)	7.03 (1.16)	<0.001
**HbA1c, %, mean (SD)**	7.32 (1.41)	7.44 (1.43)	7.10 (1.35)	<0.001
**LDL-C, mmol/L, mean (SD)**	2.38 (0.75)	2.45 (0.74)	2.25 (0.74)	<0.001
**HDL-C, mmol/L, mean (SD)**	1.15 (0.31)	1.17 (0.31)	1.10 (0.32)	<0.001
**Total cholesterol, mmol/L, mean (SD)**	4.22 (0.92)	4.32 (0.89)	4.03 (0.94)	<0.001
**Triglycerides, mmol/L, mean (SD)**	1.56 (0.88)	1.57 (0.88)	1.54 (0.88)	0.495
**Remnant cholesterol, mmol/L, mean (SD)**	0.70 (0.37)	0.70 (0.37)	0.69 (0.37)	0.477
**eGFR, mL/min/1.73 m², mean (SD)**	86.32 (26.22)	90.35 (25.50)	78.83 (25.90)	<0.001
**CRP, mg/L, mean (SD)**	2.48 (1.18)	2.25 (1.13)	2.88 (1.16)	<0.001
**Sex, n (%)**				<0.001
Male	1, 372 (45.76%)	814 (41.79%)	558 (53.14%)	
Female	1, 626 (54.24%)	1, 134 (58.21%)	492 (46.86%)	
**Smoking status, n (%)**				<0.001
Never	241 (8.04%)	179 (9.19%)	62 (5.90%)	
Former	436 (14.54%)	256 (13.14%)	180 (17.14%)	
Current	2, 321 (77.42%)	1, 513 (77.67%)	808 (76.95%)	
**Coronary heart disease (CHD), n (%)**				<0.001
No	1, 856 (61.91%)	1, 399 (71.82%)	457 (43.52%)	
Yes	1, 142 (38.09%)	549 (28.18%)	593 (56.48%)	
**Insulin use, n (%)**				0.81
No	1, 815 (60.54%)	1, 176 (60.37%)	639 (60.86%)	
Yes	1, 183 (39.46%)	772 (39.63%)	411 (39.14%)	
**Glucose-lowering medication, n (%)**				<0.001
No	541 (18.05%)	302 (15.50%)	239 (22.76%)	
Yes	2, 457 (81.95%)	1, 646 (84.50%)	811 (77.24%)	
**Antihypertensive medication, n (%)**				<0.001
No	385 (12.84%)	289 (14.84%)	96 (9.14%)	
Yes	2, 613 (87.16%)	1, 659 (85.16%)	954 (90.86%)	
**Statin use, n (%)**				0.001
No	891 (29.72%)	540 (27.72%)	351 (33.43%)	
Yes	2, 107 (70.28%)	1, 408 (72.28%)	699 (66.57%)	
**Aspirin use, n (%)**				<0.001
No	2, 297 (76.62%)	1, 615 (82.91%)	682 (64.95%)	
Yes	701 (23.38%)	333 (17.09%)	368 (35.05%)	
**CTI tertile, n (%)**				<0.001
T1 (low)	1, 000 (33.36%)	841 (43.17%)	159 (15.14%)	
T2 (medium)	999 (33.32%)	684 (35.11%)	315 (30.00%)	
T3 (high)	999 (33.32%)	423 (21.71%)	576 (54.86%)	

AF, atrial fibrillation; BMI, body mass index; CHD, coronary heart disease; CRP, C-reactive protein; DBP, diastolic blood pressure; eGFR, estimated glomerular filtration rate; FPG, fasting plasma glucose; HbA1c, glycated hemoglobin A1c; HDL-C, high-density lipoprotein cholesterol; LDL-C, low-density lipoprotein cholesterol; SBP, systolic blood pressure; TC, total cholesterol; TG, triglycerides.

### Distribution of cardiometabolic indicators by atrial fibrillation status

**Figure 2:**
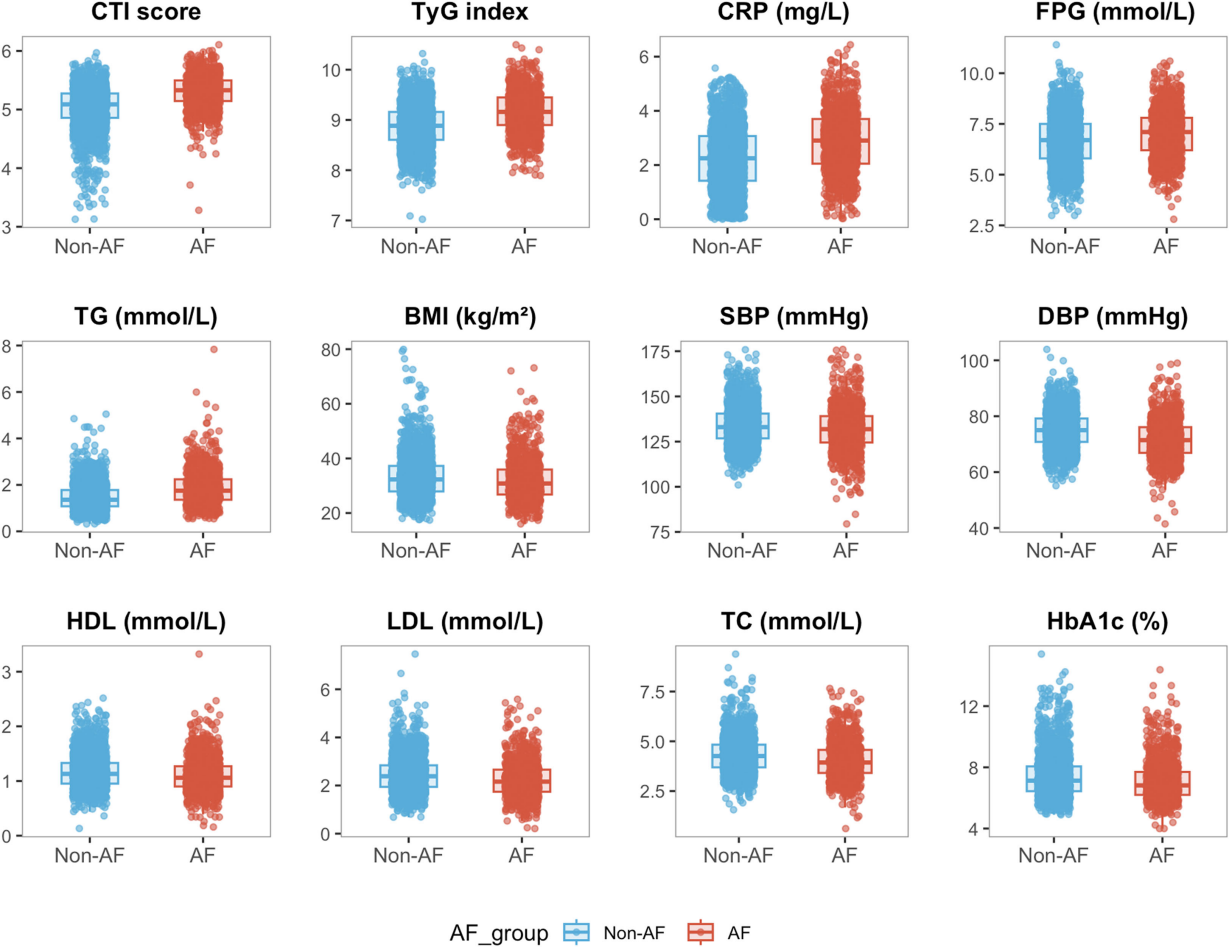
Distributions of cardiometabolic and inflammatory indicators according to AF status.

**Figure 2** presented the distributions of cardiometabolic and inflammatory indicators according to AF status. Compared with participants without atrial fibrillation, those with AF showed higher distributions of CTI score, glycemic indices, blood pressure measures, and inflammatory markers, whereas lipid parameters and body mass index exhibited partial overlap between groups.

### Multivariable logistic regression analyses for the association of continuous and three group levels of CTI with atrial fibrillation

As shown in **Table 2**, multivariable logistic regression analyses demonstrated a significant association between CTI and the risk of AF. In the unadjusted model (Model 1), CTI was strongly and positively associated with AF (continuous OR 9.81, 95% CI 7.89–12.18). This association remained statistically significant after adjustment for age, sex, and BMI in Model 2 (OR 7.73, 95% CI 6.24–9.57). After further adjustment for SBP, DBP, HbA1c, LDL-C, HDL-C, and TC in the fully adjusted model (Model 3), CTI remained independently associated with AF risk (OR 7.27, 95% CI 5.87–9.00; P < 0.001).

**Table 2:** Multivariable logistic regression analyses for the association of continuous and three group levels of CTI with AF.

Exposure	Model 1		Model 2		Model 3	
CTI (Continuous)	9.81 (7.89–12.18)	<0.001	7.73 (6.24–9.57)	<0.001	7.27 (5.87–9.00)	<0.001
CTI quartile						
T1	1 (Ref)		1 (Ref)		1 (Ref)	
T2	2.17 (1.79–2.63)	<0.001	2.15 (1.78–2.60)	<0.001	2.12 (1.75–2.57)	<0.001
T3	4.83 (4.05–5.76)	<0.001	4.32 (3.62–5.16)	<0.001	4.21 (3.52–5.03)	<0.001
P for trend	<0.0001		<0.0001		<0.0001	

Model 1: unadjusted.

Model 2: adjusted for age, sex, and BMI.

Model 3: additionally adjusted for SBP, DBP, HbA1c, LDL-C, HDL-C, total cholesterol, remnant cholesterol, eGFR, and smoking status.

BMI, body mass index; SBP, systolic blood pressure; DBP, diastolic blood pressure; HbA1c, glycated hemoglobin A1c; LDL-C, low-density lipoprotein cholesterol; HDL-C, high-density lipoprotein cholesterol; eGFR, estimated glomerular filtration rate.

When CTI was analyzed by tertiles, with the lowest tertile (T1) as the reference, the odds of AF were significantly higher in the T2 and T3 groups in Model 3, with ORs of 2.12 (95% CI 1.75–2.57) and 4.21 (95% CI 3.52–5.03), respectively. A significant linear trend was observed across CTI tertiles in all models (P for trend < 0.0001). The fully adjusted OR per SD increase was 2.07 (95% CI 1.91–2.24), remain consistent with the tertile.

### Subgroup analyses of the association between CTI and atrial fibrillation

**Figure 3:**
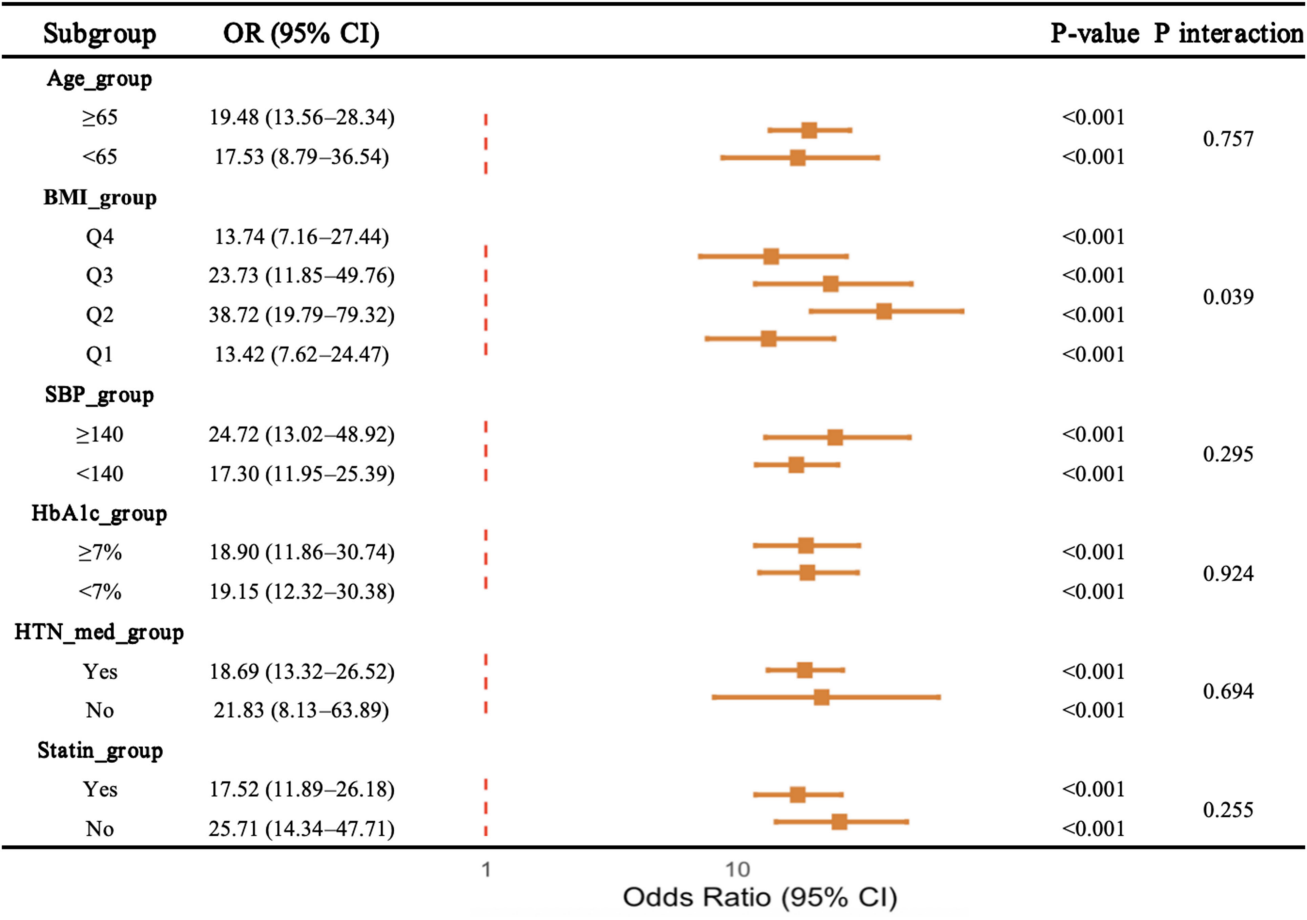
Subgroup analyses of the association between CTI and AF.

**Figure 3** presented subgroup analyses of the association between CTI and AF. Elevated CTI was consistently associated with higher odds of AF across subgroups defined by age, systolic blood pressure, HbA1c level, antihypertensive medication use, and statin use, with all subgroup specific associations remaining statistically significant.

A significant interaction was observed across body mass index quartiles, indicating heterogeneity in the magnitude of association between CTI and atrial fibrillation among BMI categories. In contrast, no significant interactions were detected for age, blood pressure status, glycemic control, antihypertensive medication use, or statin use.

### Dose-response relationship between CTI score and atrial fibrillation risk

**Figure 4:**
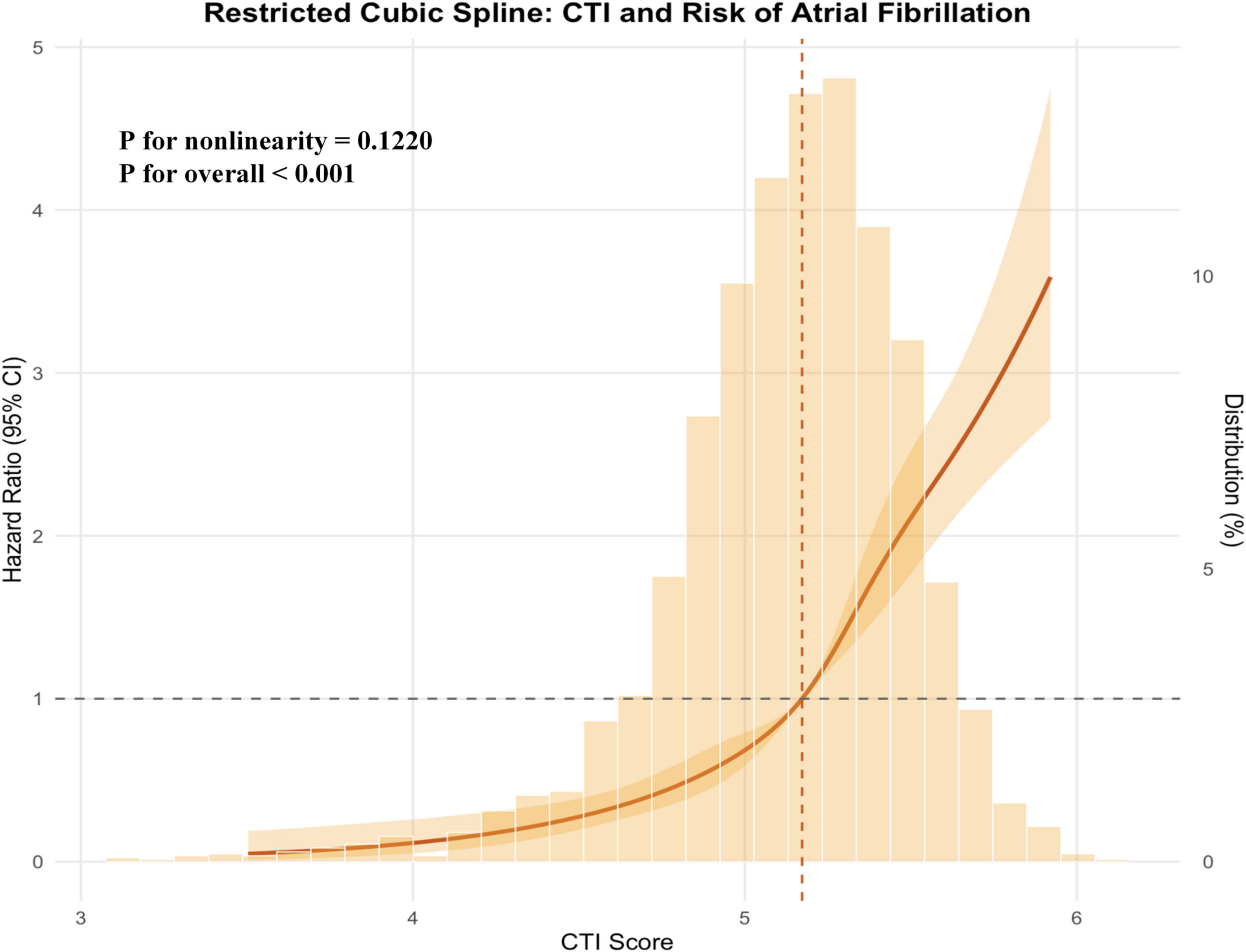
RCS curve between CTI score and the risk of AF.

**Figure 4** illustrated the dose–response relationship between CTI score and the risk of atrial fibrillation using restricted cubic spline analysis. A significant overall association between CTI and atrial fibrillation risk was observed, while no evidence of nonlinearity was detected. The risk of atrial fibrillation increased progressively with higher CTI values, with a more pronounced rise observed at higher CTI levels.

### ROC curve of CTI for atrial fibrillation

As shown in **Table 3**, adding the CTI to the fully adjusted reference model (Model 3) was associated with improved discrimination for atrial fibrillation. The C-index increased from 0.6828 to 0.7985 after inclusion of CTI, accompanied by significant improvements in reclassification metrics, including a net reclassification improvement of 0.5609 (P < 0.001) and an integrated discrimination improvement of 0.7182 (P < 0.001).

**Table 3:** Comparison of C-Index, NRI, and IDI for predictive models with CTI, CRP, and TyG in predicting atrial fibrillation (AF).

Reference model	C-Index	C-Index	NRI	P value	IDI	P value
	Basic	New		For NRI		For IDI
model 3 + CTI	0.6828	0.7985	0.5609	<0.001	0.7182	<0.001
model 3 + CRP	0.6828	0.7091	0.3575	0.005	0.5623	<0.001
model 3 + TyG	0.6828	0.7221	0.4713	<0.001	0.3387	<0.001

TyG, Triglyceride-glucose Index; CRP, C-reactive Protein; C-index, concordance index; NRI, reclassification improvement; IDI, the integrated discrimination improvement.

When CRP or TyG index was added individually to Model 3, smaller increases in the C-index were observed (0.7091 and 0.7221, respectively), with corresponding improvements in reclassification measures that were of lower magnitude than those observed with CTI.

**Figure 5:**
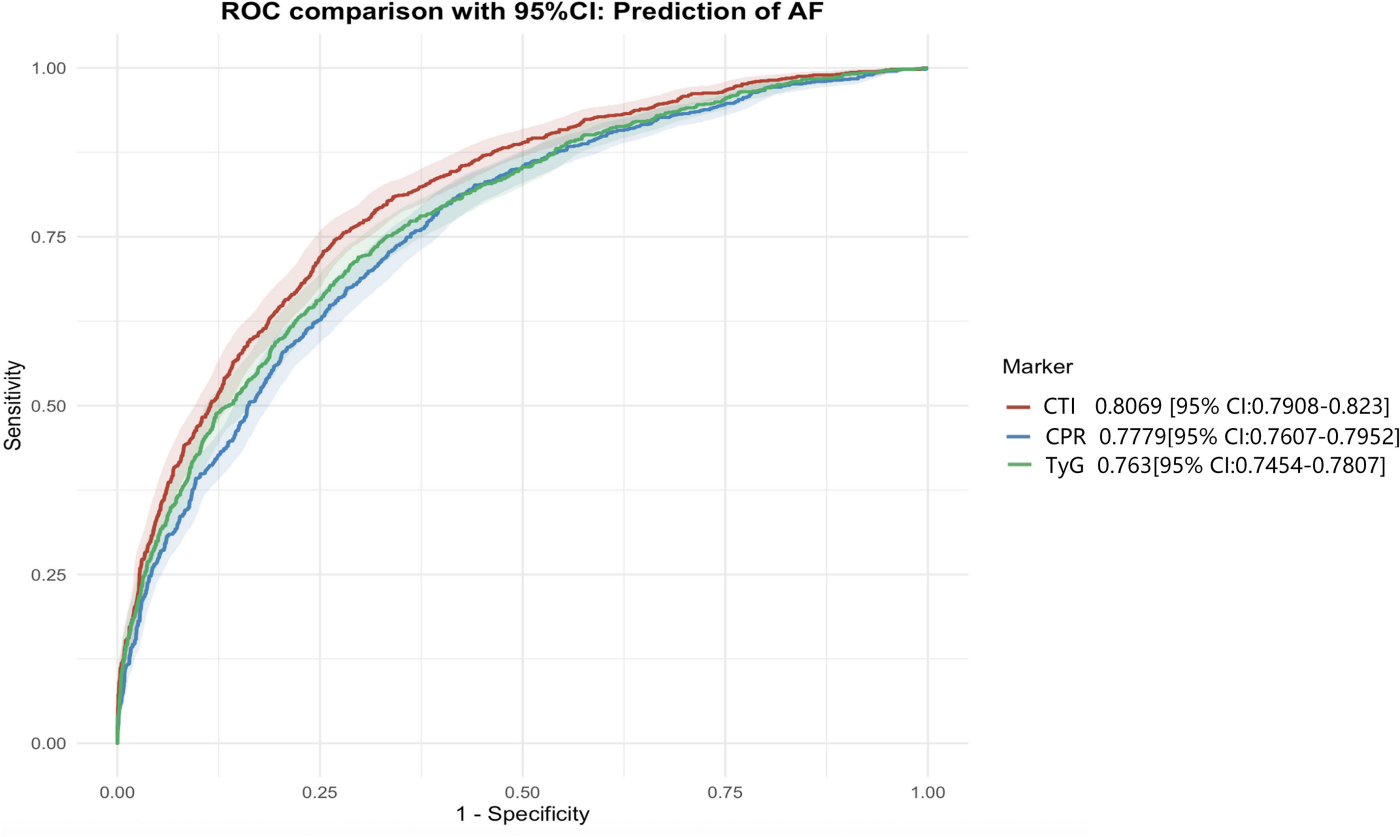
ROC for CTI and AF.

Consistent with the results shown in **Table 3**, ROC curve analyses demonstrated that CTI achieved a higher area under the curve than CRP and the TyG index (**Figure 5**).

## Discussion

This study aimed to explore the association between the CTI and AF, we demonstrated a strong and graded association between the CTI and AF risk. Higher CTI levels were consistently associated with a markedly increased odds of AF across unadjusted and fully adjusted models, with evidence of a clear dose–response relationship. Compared to CRP or the TyG index alone, CTI demonstrated superior discriminative power and classification accuracy for AF.

The superior predictive value of CTI stems from its capacity to capture the synergistic molecular mechanisms of inflammation and IR in AF development. While both components are individually associated with atrial remodeling, their interaction within a structured mechanistic framework provides a stronger biological substrate for AF. Regarding inflammation, elevated CRP reflects systemic chronic low-grade inflammation, which may activate NF-κB and NLRP3 inflammasome pathways, inducing the release of pro-inflammatory cytokines and promoting atrial fibroblast proliferation and extracellular matrix deposition (16). In parallel, altered lipid and glucose metabolism can enhance oxidative stress through mitochondrial dysfunction and reactive oxygen species generation, which has been linked to atrial myocyte injury and impaired calcium handling (17, 18). This “double-hit” mechanism collectively facilitates atrial electrical remodeling and structural remodeling (19). In this context, CTI functions similarly to other composite inflammatory indices, such as the neutrophil-to-lymphocyte ratio, which has been shown to reflect early immune activation and cardiovascular vulnerability (20). By integrating these two dimensions, CTI captures the integrated downstream effects of these interacting pathways rather than isolated metabolic abnormalities.

Numerous previous studies have explored the impact of single biomarkers on AF. For instance, several large-scale cohort studies have confirmed that high-sensitivity CRP is an independent predictor of AF onset and recurrence. Similarly, Jia et al (21). demonstrated a strong association between the TyG index and cardiometabolic risk in the Chinese population, underscoring the predictive value of metabolic dysfunction in cardiovascular diseases. Moreover, Ding et al (22). further emphasized insulin resistance as a key driver of atrial structural and electrophysiological remodeling, potentially promoting the development of atrial fibrillation through multiple metabolic pathways. However, most prior research focused on a single pathophysiological dimension. The novelty of this study lies in the construction of CTI by integrating these two indicators. This integrated approach addresses the limitations of previous studies that focused solely on either inflammation or metabolism, demonstrating that a multidimensional biomarker combination provides more precise risk stratification. It is worth noting that the performance of composite indices may vary across different cardiovascular phenotypes. For example, while the TG/HDL-C ratio has been identified as a robust marker for arterial stiffness in prediabetic populations (23), CTI may offer superior discriminative value specifically for AF due to the pivotal role of systemic inflammation in atrial remodeling. Inflammation and insulin resistance synergistically promote atrial remodeling via shared pathways (e.g., NF κB activation, NLRP3 inflammasome, oxidative stress). A multiplicative index better captures this synergy than an additive model.

Despite providing robust evidence, several limitations of this study should be noted. First, as a cross-sectional study, we could only observe an association between CTI and AF rather than establishing a causal link; prospective studies are required to clarify whether elevated CTI precedes AF onset. Second, the study relied on a single blood sample measurement and failed to capture dynamic fluctuations in the CTI index, whereas long-term trends might offer higher predictive value than a single assessment. Finally, the study population is derived from a single source with relatively homogeneous characteristics, which may limit the wider or broader scope of the findings. Fourth, the study cohort had a mean body mass index >30 kg/m² in both AF and non-AF groups, reflecting a predominantly obese population. This may limit the generalizability of our findings to leaner or more diverse populations, and the observed associations should be validated in cohorts with broader BMI distributions.

In conclusion, our findings indicate that CTI is a strong and independent marker of AF risk and outperforms traditional metabolic and inflammatory indices in relation to AF. By capturing the cumulative burden of cardiometabolic dysfunction, CTI may offer a practical and clinically meaningful tool for identifying individuals at high risk of AF. Future prospective studies are warranted to determine whether interventions targeting CTI reduction can translate into meaningful reductions in AF incidence.

## Data Availability

The original contributions presented in the study are included in the article/supplementary material. Further inquiries can be directed to the corresponding author.
